# The multiple linear regression model: to predict peak metabolic equivalents and peak oxygen pulse in patients with coronary artery disease after percutaneous coronary intervention

**DOI:** 10.3389/fcvm.2025.1459411

**Published:** 2025-04-29

**Authors:** Wenqing Xu, Yin Xiang, Bo Liu, Jianhua Yan, Tingting Zhang, Wanqi Yu, Jia Han, Shu Meng

**Affiliations:** ^1^ Department of Cardiology, Shuguang Hospital Affiliated to Shanghai University of Traditional Chinese Medicine, Shanghai, China; ^2^ Department of Rehabilitation Medicine, Shuguang Hospital Affiliated to Shanghai University of Traditional Chinese Medicine, Shanghai, China; ^3^ Department of Cardiology, Xinhua Hospital Affiliated to Shanghai Jiao Tong University School of Medicine, Shanghai, China; ^4^ Department of Rehabilitation Medicine, Sixth People's Hospital Affiliated to Shanghai Jiao Tong University School of Medicine, Shanghai, China; ^5^ College of Rehabilitation Sciences, Shanghai University of Medicine and Health Sciences, Shanghai, China

**Keywords:** coronary artery disease, cardiopulmonary exercise testing, cardiopulmonary ability, regression model, prediction equations

## Abstract

**Background:**

The clinical indicators of patients with coronary artery disease (CAD) often affect their prognosis. Cardiopulmonary Exercise Testing (CPET) can effectively evaluate the cardiopulmonary ability of CAD patients. The objective of this research was to explore the correlation between some clinical indicators and peak metabolic equivalents (peak METs) and peak oxygen pulse (O_2_P_peak_) in patients with CAD. Regression equations were further constructed for indicators with significant correlations to predict peak METs and O_2_P_peak_.

**Methods:**

152 CAD patients were recruited (M: F = 109:43, age = 64.47 ± 7.80 years, including 32 patients with chronic myocardial infarction, 46 with frailty, 93 with hypertension, and 48 with diabetes). All participants had blood biochemistry analysis, cardiac ultrasound, CPET and five time sit-to-stand (FTSTS) test. CPET was tested according to an incremental loading scheme of 10–15 w/min and peak METs, O_2_P_peak_ were recorded. Stepwise multifactorial linear regression was used to determine which clinical variables should be adjusted to improve peak METs and O_2_P_peak_.

**Results:**

Results of multifactorial linear regression showed 2 equations: peak METs = 6.768–0.116*BMI + 0.018*Hgb-0.026*age-0.005*Gensini score (Adjusted R^2^ = 0.301, F = 17.239, *p* < 0.001); O_2_P_peak_ = −1.066 + 0.264*BMI + 0.049*Hgb-0.035*age (Adjusted R^2^ = 0.382, F = 32.106, *p* < 0.001).

**Conclusion:**

BMI, Hgb, age and Gensini score can be used to predict peak METs and BMI, Hgb and age can be used to predict O_2_P_peak_ in patients with CAD clinically. Thus, tailored exercise program should be prescribed for individual CAD patient undergoing cardiac rehabilitation and modifying clinical factors such as BMI, Hgb and Gensini score will help to improve their cardiorespiratory fitness and quality of life.

## 1 Introduction

Globally, cardiovascular diseases are the leading cause of mortality (1). In 2019, an estimated 17.9 million people died from cardiovascular disease, accounting for 32% of all deaths worldwide (2). According to the *Report on Cardiovascular Health and Diseases in China 2022*, the prevalence of cardiovascular disease in China is 330 million, of which 11 million are coronary artery disease (CAD) (3). The term “CAD” describes the state of coronary artery atherosclerosis-related lumen narrowing or blockage that results in myocardial ischemia, hypoxia, or necrosis (4).

Percutaneous coronary intervention (PCI) has been the main means of achieving revascularization (5) in patients with CAD due to advancements in PCI technology and technique (6). Following PCI, cardiac rehabilitation (CR) education and prompt complication care (7) are crucial patient services linked to lowering the incidence of vascular restenosis and recurrent ischemia and enhancing quality of life (8). Therefore, following PCI, CR was advised for secondary prevention by the American Heart Association and American College of Cardiology (5). CR is a crucial strategy that can significantly improve the clinical outcomes of patients with coronary heart disease (CHD) (9–11).

Cardiopulmonary exercise testing (CPET) is a specialized exercise test that measures the exercise cardiopulmonary ability of subjects, which can non-invasively and objectively evaluate the body's response to exercise (12, 13). CPET could provide essential guidance for determining the intensity of CR in patients with CAD. Peak metabolic equivalents (peak METs) and peak oxygen pulse (O_2_P_peak_) are two important CPET indicators for assessing cardiopulmonary fitness in patients (13, 14). Cardiopulmonary fitness in patients can be effectively improved through exercise interventions (15). In some remote areas, where medical care is underdeveloped (16, 17), it is not possible to use CPET to test patients' cardiopulmonary endurance. The aim of this study is to find which clinical indicators can predict cardiopulmonary endurance indicators and to provide a solid basis for individualized CR.

Clinical indicators in patients with CAD often influence their prognosis (18). However, there are no research findings on whether these clinical indicators can be used to predict exercise cardiopulmonary endurance. Further research is needed to validate which of these indicators can reliably predict peak METs and O_2_P_peak_ in CPET and which indicator contributes the most to the predictions. Currently, it has been demonstrated that the Gensini score is an independent predictor of adverse outcomes in patients with CAD undergoing PCI (19), and the triglyceride-glucose index is a valuable predictor of prognosis in patients with premature CAD (20). Frailty is closely related to the prognosis of patients with coronary artery disease (21), but the predictive value of frailty for exercise cardiopulmonary fitness has not been standardized by research. Therefore, the design of this study also included the Essential Frailty Toolset (EFT) score and the clinical indicators included in the EFT score, and explored their predictive value for peak METs and O_2_P_peak_.

We explored the correlations between CPET and clinical indicators, such as BMI, or age-related physiological changes, such as frailty, and relevant clinical indicators including inflammatory indicators, glucose and lipid indicators, and renal function indicators. We further constructed regression equations to investigate the predictive value of clinical indicators for peak METs and O_2_P_peak_. By modulating these clinical indicators, we can achieve better peak METs and O_2_P_peak_ results. This work will be helpful for providing evidence of the clinical characteristics involving CR in CAD patients after PCI.

## 2 Method

### 2.1 Sample and procedure

The study was a data analysis that used cross-sectional data from a randomized controlled trial's recruitment phase. The objective of this research was to investigate if certain clinical indications correlated with cardiopulmonary exercise function of CAD patients undergoing PCI. Regression models were subsequently created for significantly correlated indicators in order to predict peak METs and O_2_P_peak_. The study was approved by the Ethics Committee of Xinhua Hospital Affiliated to Shanghai Jiao Tong University School of Medicine (Ethical Clearance Reference Number: XHEC-C-2020-078-1) and registered in the Chinese Clinical Registration Center (clinical trial website: http://www.chictr.org.cn/enIndex.aspx; clinical trial registry number: Chi CTR2000037435).

The study was conducted at Xinhua Hospital Affiliated to Shanghai Jiao Tong University School of Medicine. Between March 1, 2023 and June 30, 2024, a team of cardiologists and rehabilitation specialists conducted patient recruitment and data collection. Participants in the study were post-PCI patients with CAD who were hospitalized in the Department of Cardiology between March 2023 and March 2024. All participants completed a comprehensive assessment on the first day post-PCI, including blood biochemistry analysis, cardiac ultrasound, and CPET. Frailty was evaluated based on EFT. Coronary angiography was indicated for patients with: (1) typical angina symptoms unresponsive to medical therapy; (2) evidence of ischemia on non-invasive tests (such as stress echocardiography, myocardial perfusion imaging); or (3) coronary computed tomography angiography shows moderate or greater stenosis of the main branch vessels. Revascularization decisions were based on angiographic stenosis ≧70% (anatomical or intravascular ultrasound assessment) or fractional flow reserve <0.80 for intermediate lesions (40%–70%).

Patients included in this study met the following criteria: (a) chief complaint of chest pain with normal myocardial enzyme profile and troponin I; or a history of PCI; or more than 1 year post-PCI for acute myocardial infarction; (b) age ≧40 years and ≦80 years; (c) agreement to sign an informed consent form; and (d) native Chinese language. Patients with one of the following criteria were exclude from the study: (a) the occurrence of myocardial infarction within 1 year; (b) the combination of other serious diseases affecting normal movement (including cerebrovascular disease, lung disease, liver or kidney disease); (c) musculoskeletal system diseases affecting limb movement (including limb fracture or serious soft tissue injury), history of previous injury to the knee joints, or the inability to complete the sitting and standing exercises; (d) mental disorders and diseases of the brain organisms or those who refused to cooperate; (e) participation in other clinical studies in the last 3 months.

### 2.2 Measures

#### 2.2.1 General information

Social demographic characteristics of each participant were collected and recorded, including gender, age, and Body Mass Index (BMI). BMI was calculated as weight (kg) divided by height squared (m^2^), measured using a calibrated scale and stadiometer.

#### 2.2.2 Data from clinical examinations

Clinical data were examined by the hospital biochemical laboratory and extracted from the hospital's electronic medical records by the researchers. Hemoglobin (Hgb), Albumin (ALB), Glucose (GLU), Serum Total Cholesterol (TC), Triglycerides (TG), High-density lipoprotein (HDL), Low-density lipoprotein (LDL), Blood urea nitrogen (BUN), Creatinine (Cr), interleukin-6 (IL-6), interleukin-8 (IL-8), interleukin-10 (IL-10), tumor necrosis factor-α (TNF-α), N-Terminal Pro-Brain Natriuretic Peptide (NT-proBNP), and Left Ventricular Ejection Fractions (LVEF) were recorded, and the Gensini score was calculated based on the patients' coronary angiographic findings prior to stent placement.

#### 2.2.3 CPET data collection and analysis

CPET (4, 12, 22) was performed by physical therapists in the hospital's cardiopulmonary exercise room according to a standardized protocol. (1) Test equipment and program: Patients were performed using the COSMED test system (Quark PFT4 Ergo, Italy) with a power bike in 10–15 W/min power increments on the next day after PCI. (2) Environment and personnel preparation: 20–25 m^2^ treatment room; constant temperature 20–22°C, relative 50% humidity, 1 standard atmospheric pressure; equipped with 1 doctor, 1 nurse, 1 therapist (the total number of people in the treatment room shall not exceed 5). A nurse attached the electrode pads, put on the respiratory mask, sphygmomanometer, oxygen clamps, and adjusted the seat height for the subject. The therapist then conducted the test for the subject. The therapist and physician detected ECG, blood pressure, heart rate, and basal metabolism changes throughout the test. Patients were not allowed to speak throughout the test. (3) Collection of indicators: peak METs and O_2_P_peak_ values were gathered as dependent variables of the regression equation.

#### 2.2.4 EFT scores

EFT is a reliable tool for evaluating frail patients and can effectively assess the prognosis of cardiovascular disease patients (23, 24). It includes the mini-mental state examination (MMSE, 1 point for a scale score of <24), FTSTS (1 point for ≧15 s, 2 points for inability to complete it), ALB (1 point for <35 g/L) and Hgb (1 point for <130 g/L in men and <120 g/L in women), with higher EFT scores indicating deeper frailty. The five times sit-to-stand (FTSTS) test (25) was administered by a physiotherapist on the next day after PCI. A 46 cm high rigid chair without arms and with a backrest was placed against a wall. The subject was asked to perform 5 repetitions of the sit-to-stand exercise as fast as possible. The test position required the subject to sit in the center of the chair with feet on the floor, arms crossed over the chest, and legs fully extended when standing. Completion time was recorded and averaged over three repetitions, each separated by a 2 min interval, until the heart rate and blood pressure stabilized.

### 2.3 Statistical analyses

Statistical analyses were performed using SPSS 25.0 (IBM Ink., Armonk, NY) under the supervision of a medical statistician-expert. The correlation of the clinical indicators with peak METs and O_2_P_peak_ was first analyzed. The Pearson correlation analysis was used for data with a normal distribution, while the Spearman correlation test was used for data with a non-normal distribution. Indicators with significant correlations were included to build regression models with peak METs and O_2_P_peak_, respectively. Multiple linear regression modeling was employed to investigate clinical indicators associated with outcome indicators (peak METs and O_2_P_peak_).

Peak METs and O_2_P_peak_ data collected as part of the trial were included in the model as dependent variables. The variance inflation factor (VIF) of the predictor variables included in the equation was calculated to assess the presence of multicollinearity. VIF < 10 was considered to be free of multicollinearity. Additionally, goodness-of-fit analysis, ANOVA, and residual analysis were performed on the regression equation. For all analyses, *p* < 0.05 indicated statistically significant.

## 3 Results

### 3.1 Participants' characteristics

Table 1 shows general information about the participants in this study. A total of 152 patients were included in this study. Among them male: female = 109:43 and mean age was 64.47 ± 7.80, including 32 patients with chronic myocardial infarction, 46 with frailty, 93 with hypertension, and 48 with diabetes.

**Table 1:** Participants' characteristics.

Characteristic	*N* = 152
Age year, $\bar{x}\pm s$	64.47 ± 7.80
Gender, *n* (%)	
Male	109 (71.71)
Female	43 (28.29)
BMI (kg•m^−2^), $\bar{x}\pm s$	24.50 ± 3.05
Frailty, *n* (%)	
Yes	46 (30.26)
No	106 (69.74)
Chronic myocardial infarction, *n* (%)	
Yes	32 (21.05)
No	120 (78.95)
Risk factor, *n* (%)	
Hypertension	93 (61.18)
Diabetes	48 (31.58)

BMI, body mass index.

### 3.2 Correlation analysis

Table 2 shows the results of the correlation analysis. Age, BMI, Hgb, IL-8, TNF-α, NT-proBNP, Gensini score, LDL and EFT score had the significant correlations with peak METs, while age, BMI, FTSTS, Hgb, HDL, Cr and EFT score correlated significantly with O_2_P_peak_. Indicators with significant correlations were further included to build regression models with peak METs and O_2_P_peak_, respectively.

**Table 2:** Result of correlation analysis.

Indicator	peak METs	O_2_P_peak_
	CC (*P* value)	CC (*P* value)
age (year)	−0.232 (0.004)**	−0.299 (0.000)**
BMI (kg/m^2^)	−0.307 (0.000)**	0.483 (0.000)**
FTSTS (s)	−0.082 (0.316)	−0.163 (0.045)*
Hgb (g/L)	0.304 (0.000)**	0.444 (0.000)**
ALB (g/L)	0.148 (0.069)	0.066 (0.419)
IL-6 (pg/ml)	−0.126 (0.122)	−0.021 (0.799)
IL-8 (pg/ml)	−0.177 (0.030)*	0.030 (0.713)
IL-10 (pg/ml)	−0.061 (0.457)	−0.049 (0.552)
TNF-α (pg/ml)	−0.265 (0.001)**	0.061 (0.453)
NT-proBNP (pg/ml)	−0.198 (0.014)*	−0.104 (0.202)
LVEF (%)	−0.099 (0.226)	−0.148 (0.069)
Gensini score (score)	−0.231 (0.004)**	0.077 (0.347)
GLU (mmol/L)	−0.045 (0.581)	0.015 (0.858)
TC (mmol/L)	−0.121 (0.136)	−0.037 (0.651)
TG (mmol/L)	−0.060 (0.463)	0.100 (0.219)
HDL (mmol/L)	0.062 (0.448)	−0.195 (0.016)*
LDL (mmol/L)	−0.168 (0.038)*	0.015 (0.855)
BUN (mmol/L)	−0.021 (0.796)	0.110 (0.178)
Cr (umol/L)	−0.086 (0.291)	0.311 (0.000)**
EFT score (score)	−0.239 (0.003)**	−0.244 (0.002)**

Peak METs, peak metabolic equivalents; O_2_P_peak_, peak oxygen pulse; CC, correlation coefficient; BMI, body mass index; FTSTS, five time sit-to-stand; Hgb, hemoglobin; ALB, albumin; IL-6, interleukin-6; IL-8, interleukin-8; IL-10, interleukin-10; LVEF, left ventricular ejection fractions; GLU, glucose; TC, serum total cholesterol; TG, triglycerides; HDL, high-density lipoprotein; LDL, low-density lipoprotein; BUN, blood urea nitrogen; Cr, creatinine; EFT, essential frailty toolset.

* *P* < 0.05.

** *P* < 0.01.

### 3.3 Peak METs regression model

#### 3.3.1 Results of the peak METs regression equation

Multiple linear regression equations were constructed in a stepwise manner with age, BMI, Hgb, IL-8, TNF-α, NT-proBNP, Gensini score, LDL and EFT score as independent variables and peak METs as the dependent variable. It was found that BMI, Hgb, age and Gensini score could be included as predictor variables in the regression equation and the rest of the indicators were excluded (Table 3). The final equation was:

$$\begin{array}{rl} & \mathrm{peak}\;\mathrm{METs}=6.768\text{-}0.116*\mathrm{BMI}+0.018*\mathrm{Hgb}\text{-}0.026*\mathrm{age}\text{-}0.005*\\ & Gensini\;score\;(\mathrm{Adjusted}\;R^{2}=0.301,F=17.239,\;p\;\;<\;0.001); \end{array}$$

**Table 3:** Peak METs regression model.

Model 1	Unstandardized coefficient		Standardized coefficient	*t*	Significance	VIF
	B	Standard Errors	Beta			
(Constant)	6.768	1.003		6.750	0.000	
BMI (kg/m^2^)	−0.116	0.020	−0.414	−5.880	0.000	1.069
Hgb (g/L)	0.018	0.004	0.308	4.413	0.000	1.054
Age (year)	−0.026	0.008	−0.240	−3.361	0.001	1.100
Gensini score (score)	−0.005	0.002	−0.184	−2.648	0.009	1.042

Implicit variable: peak METs.

Peak METs, peak metabolic equivalents; VIF, variance inflation factor; BMI, body mass index; Hgb, hemoglobin.

There was a statistically significant difference in the effect of different BMI on peak METs (*b* = −0.116, *t* = −5.880, *P* < 0.001), implying that for every 1 kg/m^2^ increase in BMI, there was a 0.116 decrease in peak METs. The effect of different Hgb on peak METs was statistically different (*b* = 0.018, *t* = 4.413, *P* < 0.001), implying that for every 1 g/L increase in Hgb, there was a 0.018 increase in peak METs. There was a statistically significant difference in the effect of different age on peak METs (*b* = −0.026, *t* = −3.361, *P* = 0.001), implying that for every 1-year increase in age, there was a 0.026 decrease in peak METs. The effect of different Gensini score on peak METs was statistically different (*b* = −0.005, *t* = −2.648, *P* = 0.009), implying that for every 1-score increase in Gensini score, there was a 0.005 decrease in peak METs.

#### 3.3.2 Analysis of peak METs regression equations

The regression equation of peak METs was analyzed in terms of multicollinearity, goodness of fit, ANOVA and variance residuals.

VIFs for these four variables' VIFs were all well below 10, so there was no multicollinearity among them. This linear regression model had a good fit (adjusted R^2^ = 0.301), which means that the results of this operation can reliably reflect the effect of BMI, Hgb, age and Gensini score on peak METs. The regression equation was significant, F = 17.239, *p* < 0.001, implying that at least one of the four independent variables can significantly affect the dependent variable. (Tables 3, 4).

**Table 4:** Peak METs regression model summaries.

Model	Adjusted R^2^	Durbin-Watson	F	*P*
1	0.301	1.500	17.239	<0.001

Predictor variables: (constants), BMI, Hgb, age, Gensini score; Implicit variable: peak METs.

Peak METs, peak metabolic equivalents.

**Figure 1:**
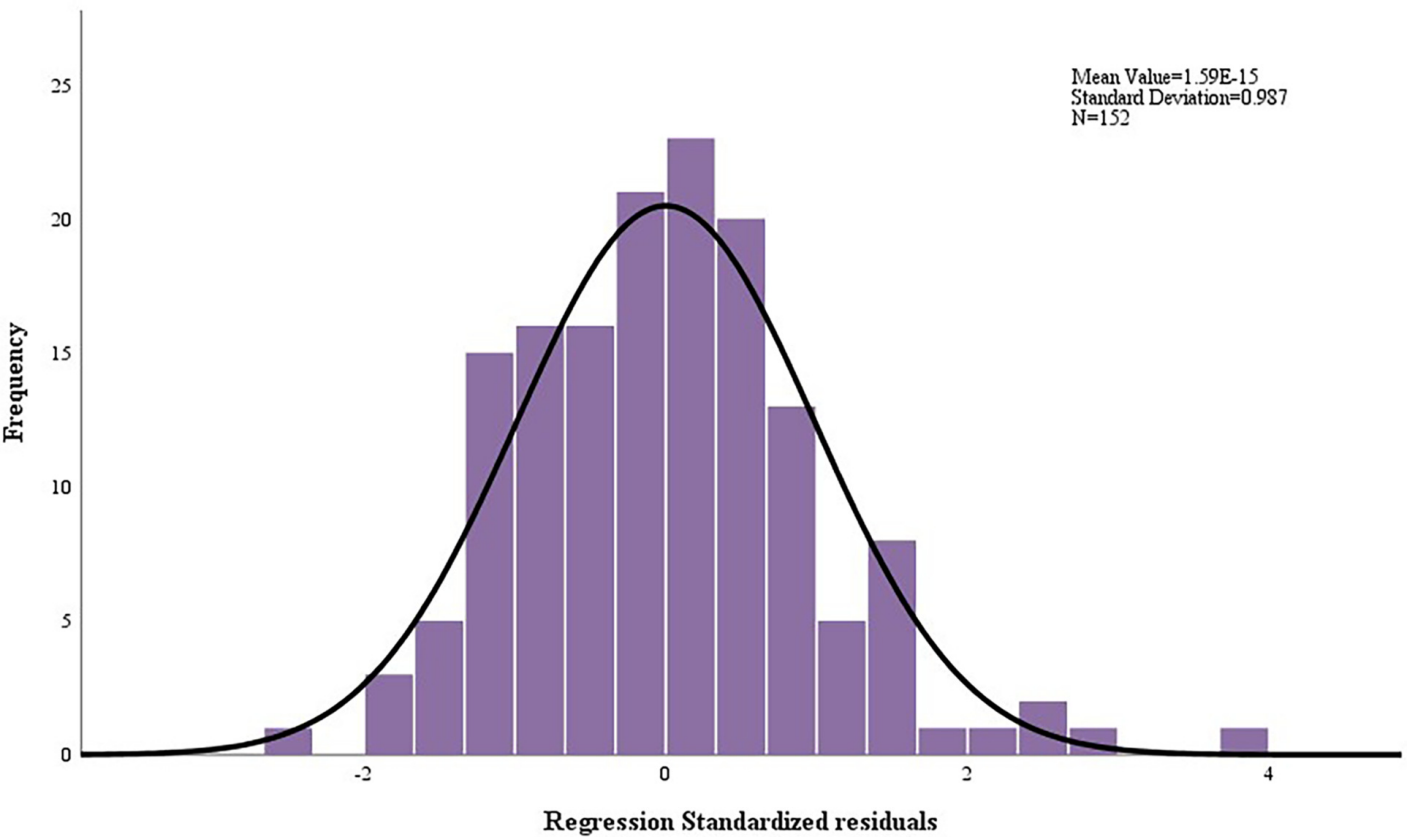
Histogram of residuals for peak METs regression model.

**Figure 2:**
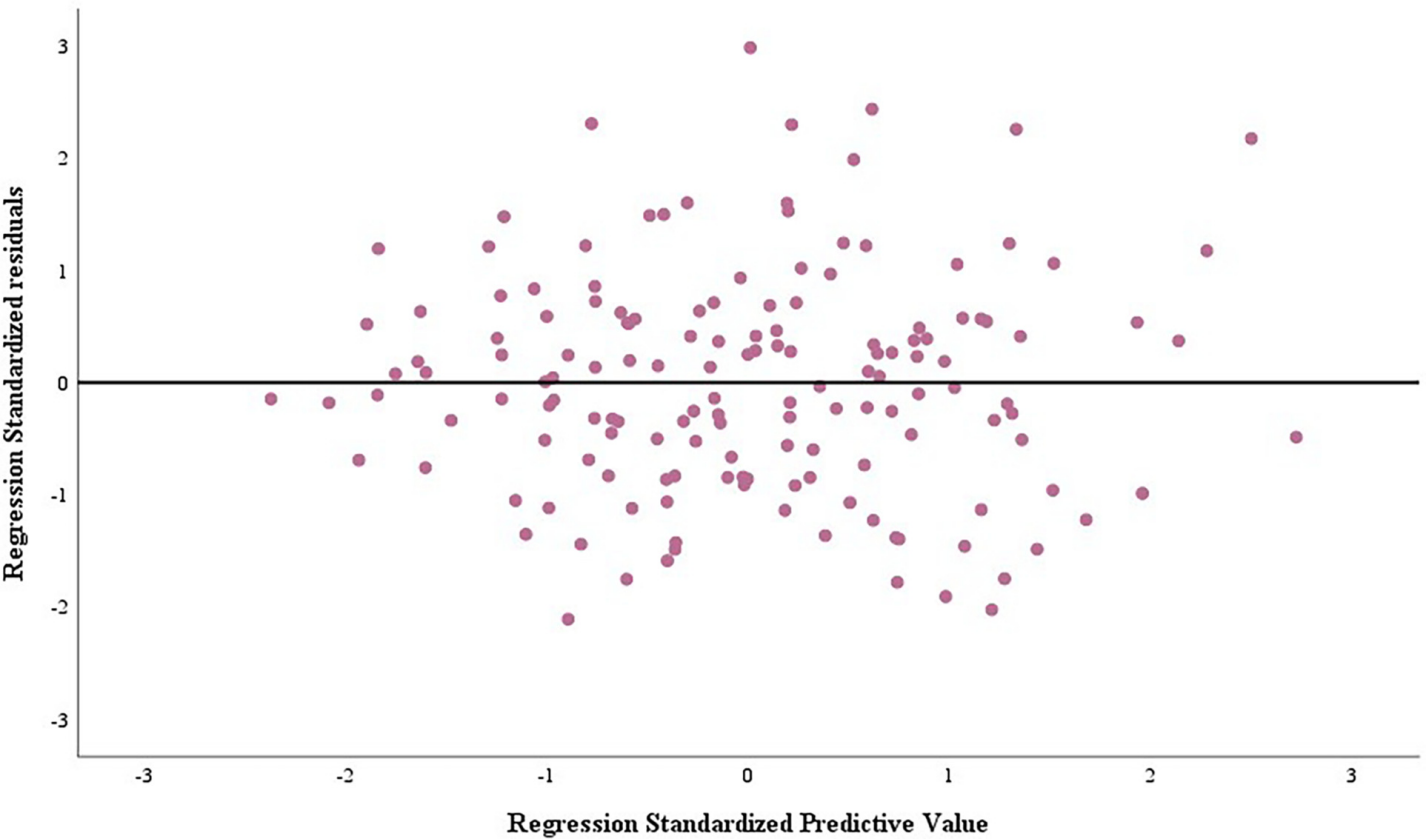
Scatterplot of residuals for peak METs regression model.

Residual histograms (Figure 1) and residual plots (Figure 2) demonstrated that the residuals were normally distributed, standardized around the value of 0, symmetrically distributed above and below 0, and that residual normality, variance chi-square, and independence were satisfied.

### 3.4 O_2_P_peak_ regression model

#### 3.4.1 Results of the O_2_P_peak_ regression equation

Multiple linear regression equations were constructed in a stepwise manner using age, BMI, FTSTS, Hgb, HDL, Cr and EFT score as independent variables and O_2_P_peak_ as the dependent variable. It was found that BMI, Hgb and age could be included as predictor variables in the regression equation and FTSTS, HDL, Cr and EFT score was excluded (Table 5). The final equation was:

$$\begin{array}{rl} O_{2}P_{\mathrm{peak}}= & \;\;-1.066+0.264*\mathrm{BMI}\\ & +0.049*\mathrm{Hgb}-0.035*\mathrm{age}\;\\ & \;\;(Adjusted\;R^{2}=0.382,\;F=32.106,\;p<0.001); \end{array}$$

**Table 5:** O_2_p_peak_ regression model.

Model 2	Unstandardized coefficient		Standardized coefficient	*t*	Significance	VIF
	B	Standard Errors	Beta			
(Constant)	−1.066	2.106		−0.506	0.613	
BMI (kg/m^2^)	0.264	0.042	0.413	6.250	0.000	1.066
Hgb (g/L)	0.049	0.009	0.360	5.540	0.000	1.031
age (year)	−0.035	0.017	−0.139	−2.086	0.039	1.080

Implicit variable: O_2_P_peak._

O_2_P_peak_, peak oxygen pulse; VIF, variance inflation factor; BMI, body mass index; Hgb, hemoglobin.

There was a statistically significant difference in the effect of different BMI on O_2_P_peak_ (*b* = 0.264, *t* = 6.250, *p* < 0.001), implying that for every 1 kg/m^2^ increase in BMI, there was a 0.264 increase in O_2_P_peak_. The effect of different Hgb was statistically different in O_2_P_peak_ (*b* = 0.049, *t* = 5.540, *p* < 0.001), implying that each 1 g/L increase in Hgb was associated with a 0.046 increase in O_2_P_peak_. The effect of different age was statistically different in O_2_P_peak_ (*b* = −0.035, *t* = −2.086, *p* = 0.039), implying that each 1-year increase in age was associated with a 0.035 decrease in O_2_P_peak_.

#### 3.4.2 Analysis of O_2_P_peak_ regression equations

The regression equation of O_2_P_peak_ was analyzed in terms of multicollinearity, goodness of fit, ANOVA and variance residuals.

There was no multicollinearity between the four variables, since all four VIFs were less than 10. This linear regression model had a good fit (R^2^ = 0.382), which means that the results of this operation can reliably reflect the effect of BMI, Hgb and age on O_2_P_peak_. The regression equation was significant, F = 32.106, *p* < 0.001, indicating that at least one of the four independent variables can significantly affect the dependent variable (Tables 5, 6).

**Table 6:** O_2_p_peak_ regression model summaries.

Model	Adjusted R^2^	Durbin-Watson	F	*P*
2	0.382	1.699	32.106	<0.001

Predictor variables: (constants), BMI, Hgb, age; Implicit variable: O_2_P_peak._

**Figure 3:**
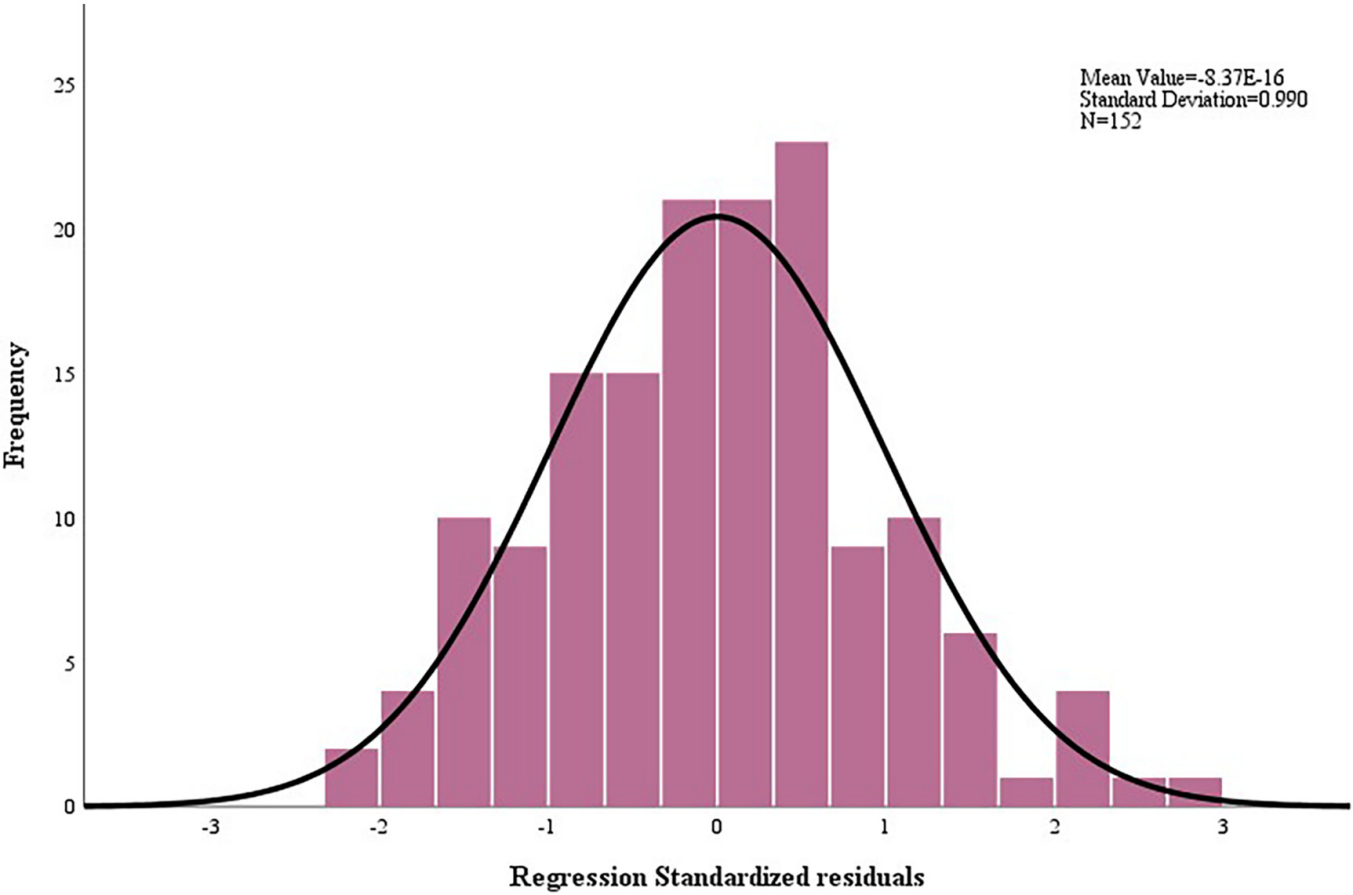
Histogram of residuals for O_2_P_peak_ regression model.

**Figure 4:**
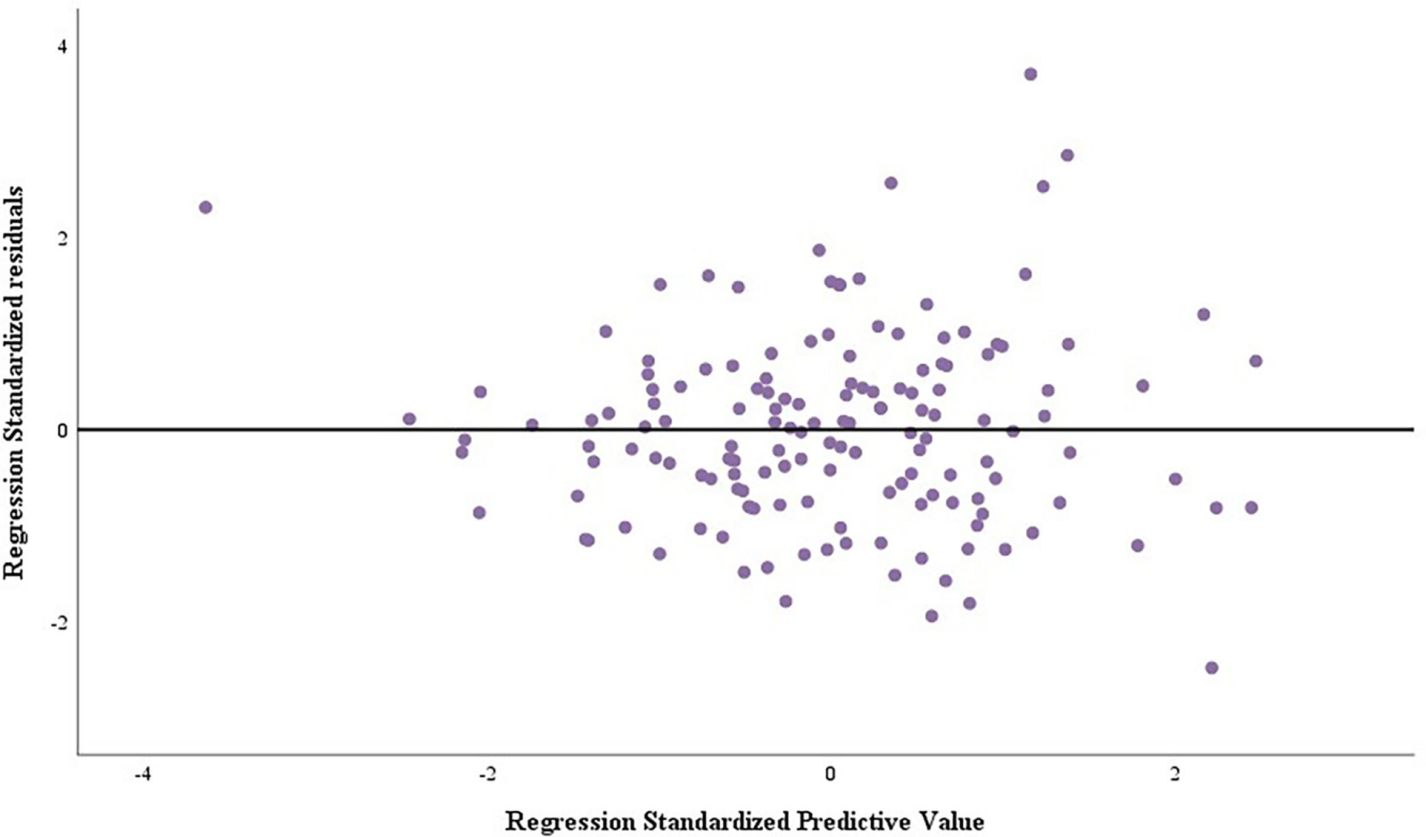
Scatterplot of residuals for O_2_P_peak_ regression model.

Residual histograms (Figure 3) and residual plots (Figure 4) demonstrated that the residuals were normally distributed, standardized around the value of 0, symmetrically distributed above and below 0, and that residual normality, variance chi-square, and independence were satisfied.

### 3.5 Intersection of BMI in two regression equations

The results of the 2 equations showed that peak METs decreased and O_2_P_peak_ increased as BMI increased. Thus, we found the value of BMI at the intersection of the two equations, controlling for the rest of the variables, by using the predicted quantities of BMI from the two equations as well as the constants. The equations for finding the value are as follows: (1) 6.768–0.116*BMI = −1.066 + 0.264*BMI; (2) BMI = 20.616 kg/m^2^.

## 4 Discussion

We have demonstrated that BMI, Hgb, age and Gensini score can be used to predict peak METs and BMI, Hgb and age can be used to predict O_2_P_peak_ in patients with CAD clinically. Regression equation for predicting peak METs and O_2_P_peak_ are as follows: peak METs = 6.768–0.116*BMI + 0.018*Hgb-0.026*age-0.005*Gensini score; O_2_P_peak_ = −1.066 + 0.264*BMI + 0.049*Hgb-0.035*age. Thus, appropriate weight and nutritional status management guidelines, individualized exercise prescription for patients of different ages, and timely vascular status assessment and treatment, will be helpful to overcome frailty and improve exercise capacity and quality of life for CAD patients undergoing exercise-based cardiac rehabilitation.

In this study, we found that peak METs decreased and O_2_P_peak_ increased as BMI increased. The effect of BMI on exercise cardiorespiratory fitness was controversial. The effect of BMI on prognosis is not purely linear. For example, BMI had J-shaped associations with overall mortality and most specific causes of death (26). Previous study found O_2_P_peak_ was higher in the obese group; nevertheless, this variable became significantly lower if the ratio between O_2_ pulse and kilogram fat-free body mass or kilogram body weight were taken into consideration (27). Another study also confirmed that in patients with obesity, absolute aerobic power increased with increasing BMI, while the maximal aerobic capacity relative to body weight (O_2_P_peak_/kg) significantly reduced. Conversely, an inverse effect was detectable after bariatric surgery, when absolute aerobic power slightly decreased and functional capacity significantly increased (28).

The following explanations could be rational for BMI's involvement in O_2_P_peak_. Obese subjects use a greater amount of O_2_ to accomplish an equal external work load when compared to normal subjects because their increased body mass requires a greater metabolic energy exchange. Nevertheless, O_2_P_peak_/kg is the parameter that really reflects the ability to carry out daily activities and represents the strongest long-term prognostic marker for both disability and mortality. Previous studies in pathological obesity demonstrated an improvement in gas exchange (29) as well as left-ventricular morphology and function during exercise after weight reduction (30).

So maintaining an appropriate BMI is essential for improved cardiorespiratory fitness. With the two multiple linear regression equations obtained, we could obtain an optimal BMI value (20.616 kg/m^2^) that is likely to keep a relatively good level of both peak METs and O_2_P_peak_.

In this study, Hgb was significantly correlated with peak METs and O_2_P_peak_, and was also a strong predictor of peak METs and O_2_P_peak_ in the final constructed regression equation. Hgb level tends to affect people's exercise endurance due to its oxygen transport capacity, which explains its correlation with cardiopulmonary function indexes. Therefore, in clinical practice, we may use Hgb to reasonably predict patients' peak METs and O_2_P_peak_. Hgb is also one of the key indicators in the EFT score. This study also attempted to explore the correlation of EFT scores with peak METs and O_2_P_peak_. EFT is known to exhibit better predictive ability than other frailty scales (31). This study demonstrated a significant correlation between EFT score and cardiopulmonary exercise indicators, but multiple stepwise linear regression analysis excluded it from the equation as a predictor of peak METs and O_2_P_peak_. The conjecture for the failure to find a correlation with EFT is that the sample size is insufficient. The majority of CAD patients after PCI in this study had an EFT score of 1 or 2, resulting in a lack of patients with higher EFT scores, unlike CHD patients after Coronary Artery Bypass Grafting (CABG) (24). Thus, trials with larger sample size are needed in the future.

The results of this study showed that age was a reasonable predictor of peak METs and O_2_P_peak_ in patients with CAD, with peak METs decreasing by 0.26 and O_2_P_peak_ decreasing by 0.35 for every ten years of age. Peak METs adequately reflects the patient's tolerance of exercise intensity, and O_2_P_peak_ accounts for maximum heart rate, which is a common indicator for setting up exercise intensity in the exercise prescriptions (32). Therefore, older individuals should adopt a lower exercise intensity in exercise prescriptions than younger individuals to prevent cardiovascular events during exercise rehabilitation (33, 34). Correspondingly, older patients may need to exercise for longer periods of time to achieve similar benefits as younger patients.

This study also discovered that Gensini score can predict peak METs in CAD patients, which proves that the degree of coronary artery stenosis affects the cardiopulmonary fitness. A study noted that patients who developed ST-segment depression during the active phase of the exercise test had a higher Gensini score compared to patients who developed ST-segment depression only during the recovery phase (35). This also demonstrates the predictive value of the Gensini score for exercise capacity in patients with CAD. In clinical practice, we need to pay close attention to the coronary artery stenosis of patients. When necessary, we need to recommend patients to undergo PCI as early as possible to avoid further decline in cardiopulmonary fitness.

There were some limitations in this study. It is a single-center cross-sectional study. The results may have certain limitations and need further validation and generalization involving more participants and more medical institutions. In addition, this study did not measure cardiovascular events during exercise rehabilitation in post-PCI patients. Prospective research is needed to explore methods for predicting cardiovascular events during follow-up of exercise-based cardiac rehabilitation in patients with CAD.

## 5 Conclusion

BMI, Hgb, age and Gensini score can be used to predict peak METs and BMI, Hgb and age can be used to predict O2Ppeak in patients with CAD clinically. Thus, individually tailored exercise program should be prescribed for CAD patients undergoing cardiac rehabilitation. Meanwhile, modifying clinical factors such as BMI, Hgb and Gensini score will help to improve their cardiorespiratory endurance, exercise capacity and quality of life.

## Data Availability

The raw data supporting the conclusions of this article will be made available by the authors, without undue reservation.
